# A Case of a Cardiac Re-Rupture Caused by the Removal of Pericardial Patches Late after a Sutureless Repair for a Cardiac Rupture

**DOI:** 10.70352/scrj.cr.26-0414

**Published:** 2026-10-02

**Authors:** Rena Usui, Shunsuke Nakata, Shiori Kako, Hideki Ito, Masato Mutsuga, Akihiko Usui

**Affiliations:** 1 Department of Cardiovascular Surgery, Fujita Health University Okazaki Medical Center, Okazaki, Aichi, Japan; 2 Department of Cardiac Surgery, Nagoya University Graduate School of Medicine, Nagoya, Aichi, Japan

**Keywords:** cardiac rupture, pseudoaneurysm, sutureless repair, myocardial infarction

## Abstract

**INTRODUCTION:**

Surgical treatment is the only reliable therapeutic option for a left ventricular free wall rupture (LVFWR). There are 2 major surgical techniques, namely, sutureless repair and sutured repair; a ventricular pseudoaneurysm is a late postoperative complication. We herein report a case of a re-ruptured left ventricular pseudoaneurysm after a sutureless bovine pericardial patch repair.

**CASE PRESENTATION:**

The patient was a 79-year-old woman who underwent a sutureless pericardial patch repair for a blow-out-type left ventricular rupture following an anterior myocardial infarction 10 months previously. She was referred to Fujita Health University Okazaki Medical Center with a diagnosis of thoracic empyema and a suspected pericardial patch infection. Pleural effusion was positive for a few *Streptococcus agalactiae*. Coronary CT revealed the occlusion of the proximal left anterior descending branch and aneurysmal dilatation of the anterolateral wall of the left ventricle covered with pericardial patches. The operation was performed via a left fifth intercostal thoracotomy without cardiopulmonary bypass. The covering bovine pericardial patches and felt strips were easily removed, and the omental flap was secured in place. However, an emergency reoperation was necessitated for a left hemothorax 3 hours after surgery. Active bleeding was observed from the aneurysmal area, which had been previously covered with pericardial patches. After inducing deep hypothermia of 20°C, the fragile tissue surrounding the bleeding site was debrided under ventricular fibrillation. A 3 × 3-cm myocardial tissue defect was observed and closed using a bovine pericardial patch (Model 4700; Edwards Lifesciences, Irvine, CA, USA) with ten 2-0 MH U sutures (ETHIBOND EXCEL polyester suture, 2-0, MH needle, X843H; Ethicon, Somerville, NJ, USA). All intraoperative cultures were negative, and the fragile tissue revealed an organizing hematoma lacking any myocardial tissue. She experienced prolonged respiratory failure, requiring tracheostomy and mechanical ventilation for 6 months, and was moved to the rehabilitation ward, but died 1 year after surgery due to pneumonia.

**CONCLUSIONS:**

Left ventricular pseudoaneurysm formation is a notable late complication that should be carefully monitored during the postoperative follow-up after surgery for LVFWR. We encountered a case of a re-ruptured left ventricular pseudoaneurysm after a sutureless bovine pericardial patch repair. The possibility of re-rupture must be considered because the myocardial tissue beneath the pericardial patch might disappear and be replaced with an organizing hematoma.

## Abbreviations

CPB
: cardiopulmonary bypass
LVFWR
: left ventricular free wall rupture

## INTRODUCTION

An LVFWR is a fatal complication of acute myocardial infarction. Surgical treatment is the only reliable therapeutic option for LVFWR. However, an optimal surgical technique has not yet been established. There are 2 major surgical techniques for LVFWR: one is to cover the ruptured area with patches using a surgical adhesive, such as BioGlue Surgical Adhesive (Century Medical, Tokyo, Japan) or something similar, and the other is to suture a patch to the ruptured area. The former is called a sutureless repair, and the latter is called a sutured repair. The outcomes and follow-up results of each technique remain unclear because of their rarity. However, a left ventricular pseudoaneurysm has been reported as a late postoperative complication after LVFWR in a few cases in the literature.^1,2)^ We herein report a case of a re-ruptured left ventricular pseudoaneurysm after a sutureless bovine pericardial patch repair.

## CASE PRESENTATION

The patient was a 79-year-old woman who underwent a sutureless pericardial patch repair for a blow-out-type left ventricular rupture following an anterior myocardial infarction 10 months previously at another hospital. Surgery was performed via median sternotomy with extracorporeal membrane oxygenation support. The blow-out-type ventricular rupture in the diagonal artery area was covered with bovine pericardial patches using BioGlue and TachoSil Tissue Sealant Patch (CSL Behring, Tokyo, Japan) and it was completed with several felt strip stay sutures. At a local clinic follow-up, left pleural effusion and elevated inflammatory markers were observed. Despite drainage of the pleural effusion with antibiotic therapy, no improvement was observed. She was thereafter referred to Fujita Health University Okazaki Medical Center with a diagnosis of thoracic empyema and a suspected patch infection.

Her clinical findings were as follows: body temperature of 37.7°C, blood pressure of 130/50 mmHg, heart rate of 95 beats/min with normal sinus rhythm, and SpO2 of 98% under 1 L/min of nasal oxygen. She had a left chest drainage tube placed, and no murmur was observed. She also demonstrated decreased breath sounds in the left lower part of the lung and mild leg edema. A laboratory investigation showed high inflammatory markers, including a white blood cell count of 11.2/μL and a C-reactive protein level of 7.99 mg/dL, and a slightly low hemoglobin level (9.7 g/dL) without elevated cardiac enzymes (aspartate aminotransferase 18 U/L, creatine kinase 31 U/L). Chest radiography revealed left pleural effusion with septations. The pleural effusion from the chest drain was serous and positive for a few *Streptococcus agalactiae* only in the initial drainage sample. Electrocardiography revealed a QS pattern at V1–4. CT revealed pericardial effusion and fluid collection at the inferior margin of the median sternotomy wound. Coronary CT demonstrated complete occlusion of the proximal left anterior descending artery and aneurysmal dilatation of the anterolateral wall of the left ventricle covered with pericardial patches and felt strips (**Fig. 1**). Transthoracic echocardiography also demonstrated an aneurysm of the anterolateral wall and akinesis of the anterior wall of the left ventricle with a left ventricular ejection fraction of 40% (**Fig. 2**).

**Fig. 1:**
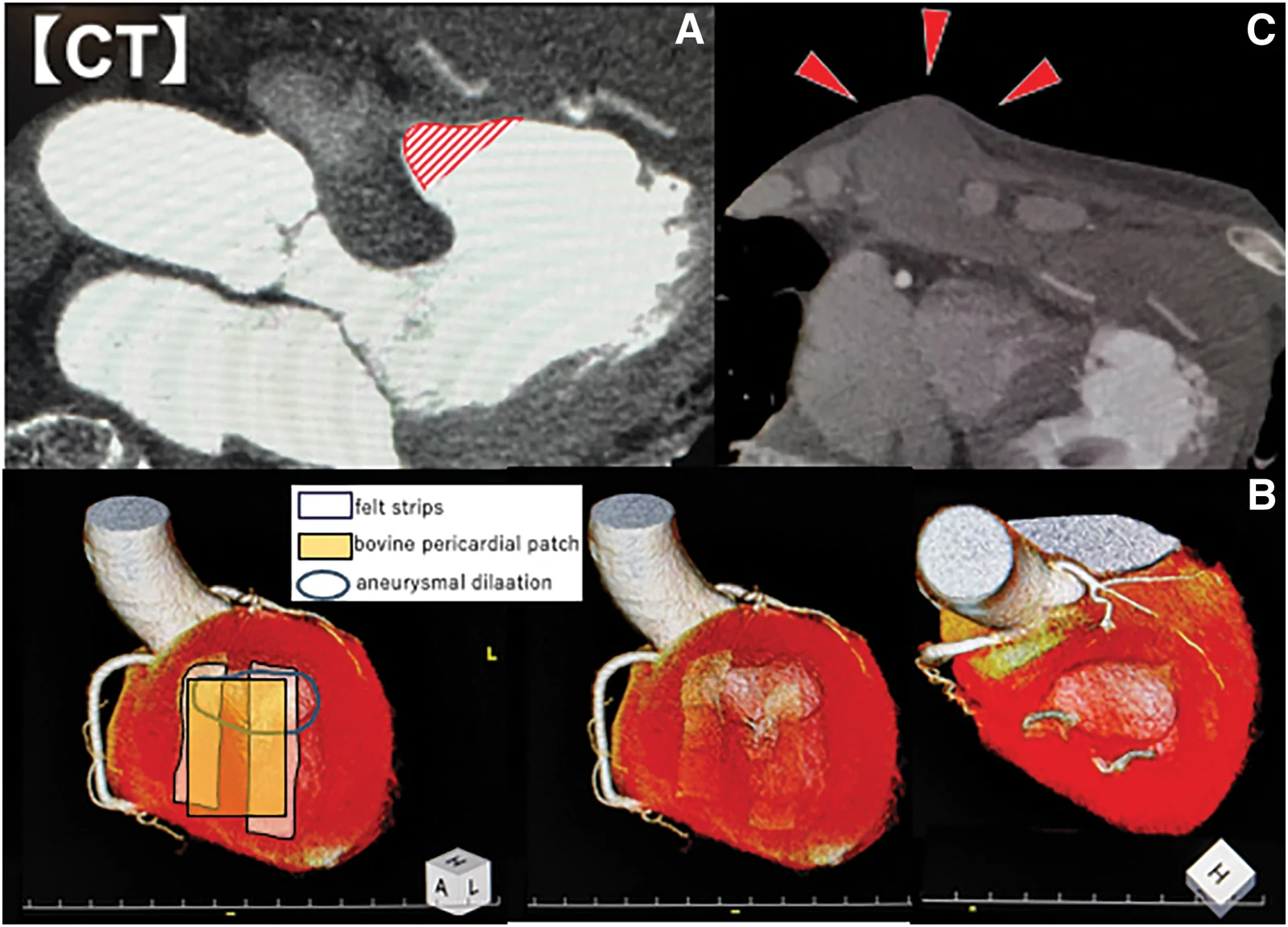
Coronary CT on admission. (**A**) The area marked with red diagonal lines demonstrates aneurysmal dilatation covered with patches and felt strips. (**B**) 3D CT revealed complete occlusion of the proximal left anterior descending artery and aneurysmal dilatation located cranial to the felt strips. (**C**) Pericardial effusion and fluid collection at the inferior margin of the median sternotomy wound. Red arrowheads show fluid collection.

**Fig. 2:**
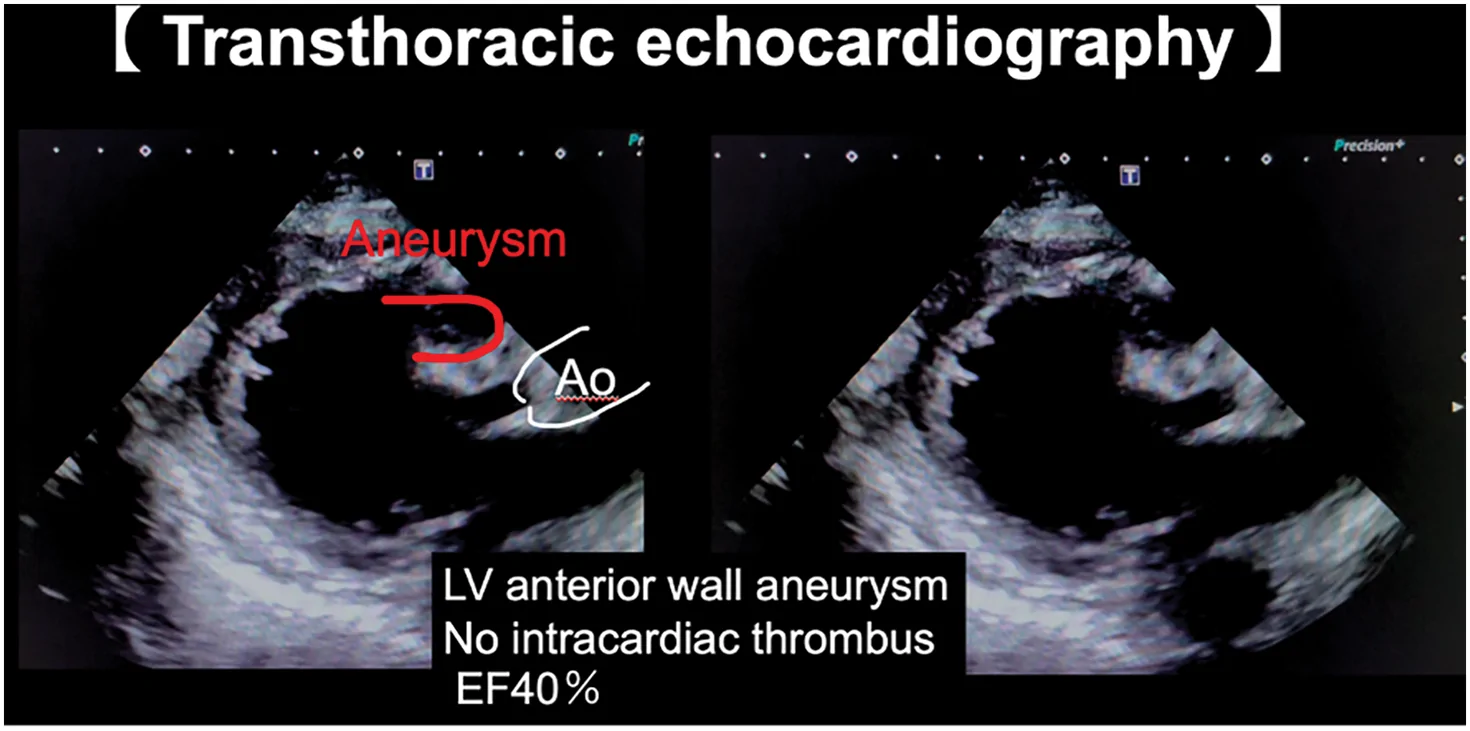
Transthoracic echocardiography demonstrated an aneurysm of the anterior wall. Ao, aorta; EF, ejection fraction; LV, left ventricle

Although thoracoscopic curettage of the abscess cavity and pericardial drainage were performed, no improvement in either the inflammatory markers or pleural/pericardial effusion was observed. Therefore, the removal of the covering pericardial patches and omental flap was planned for the diagnosis of pericardial patch infection.

The operation was performed via a left fifth intercostal thoracotomy without CPB to avoid sternal osteomyelitis. Upon opening the pericardium, turbid pericardial effusion was noted. The covering bovine pericardial patches and felt strips were easily removed, and the surface under the patches revealed reddish-white aneurysmal tissue (**Fig. 3**). Subsequently, the omental flap was harvested through the lower edge of the median incision and placed over the left ventricular aneurysm, and the initial procedure was completed. The operative time was 173 minutes.

**Fig. 3:**
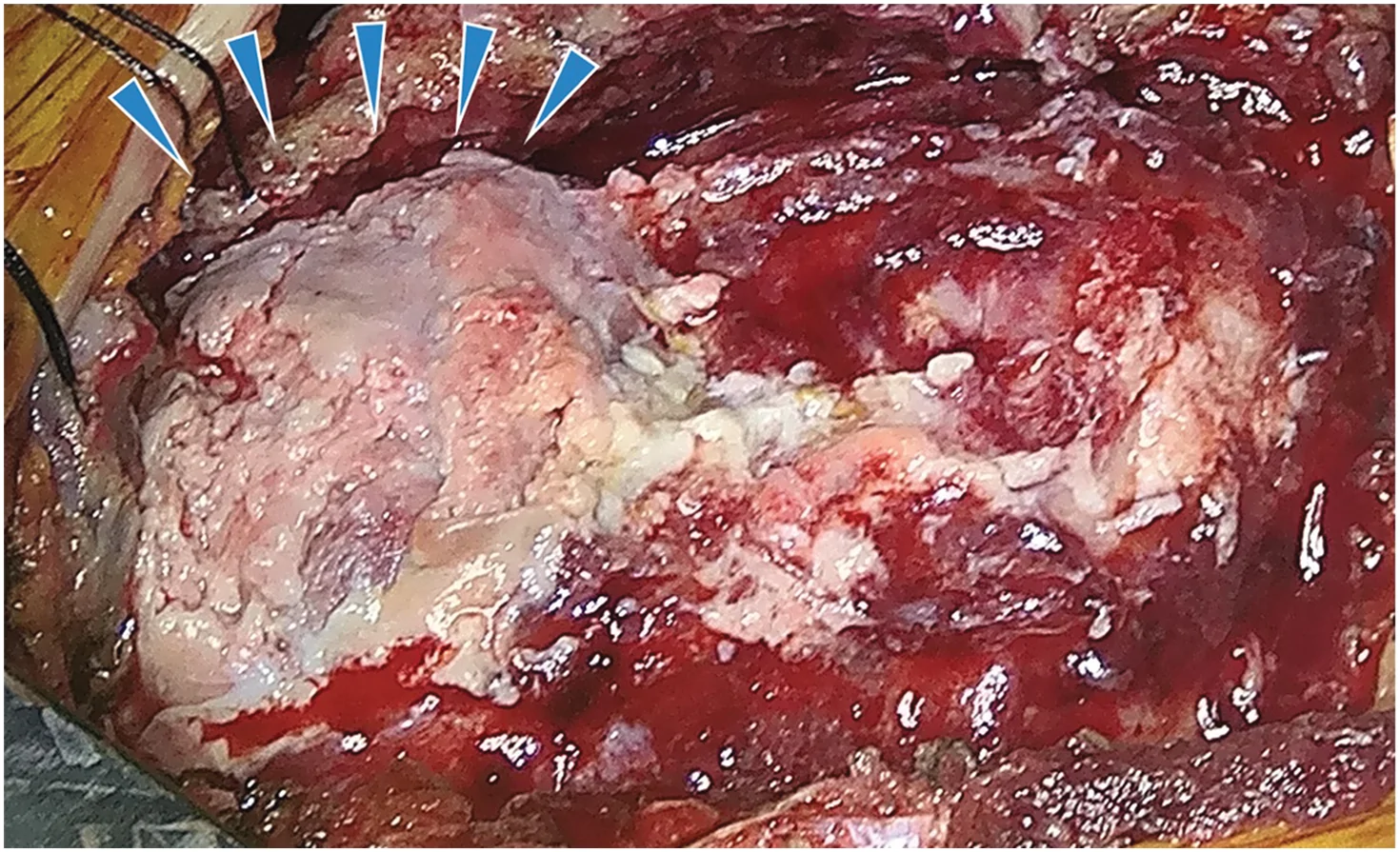
An aneurysmal change was identified under the bovine pericardial covering. Blue arrowheads show an aneurysmal change.

However, 3 hours postoperatively, the patient developed sudden hypotension and a left hemothorax, necessitating an emergency reoperation. CPB was established through the right femoral artery and vein. Upon reopening the chest, active bleeding was observed from the aneurysmal area, which had been previously covered with pericardial patches (**Fig. 4**). After inducing deep hypothermia with a nasopharyngeal temperature of 20°C, the fragile tissue surrounding the bleeding site was debrided under ventricular fibrillation, and a 3 × 3-cm myocardial tissue defect was observed (**Fig. 5**). The defect was closed using a 4 × 4-cm bovine pericardial patch (Model 4700; Edwards Lifesciences, Irvine, CA, USA) with 2-0 MH U sutures (ETHIBOND EXCEL polyester suture, 2-0, MH needle, X843H; Ethicon, Somerville, NJ, USA). A left chest tube was placed (**Fig. 6**). CPB was successfully weaned after defibrillation, and the surgery was completed with an intra-aortic balloon pump in place. The operative time was 315 minutes, with CPB and ventricular fibrillation times of 185 and 114 minutes, respectively.

**Fig. 4:**
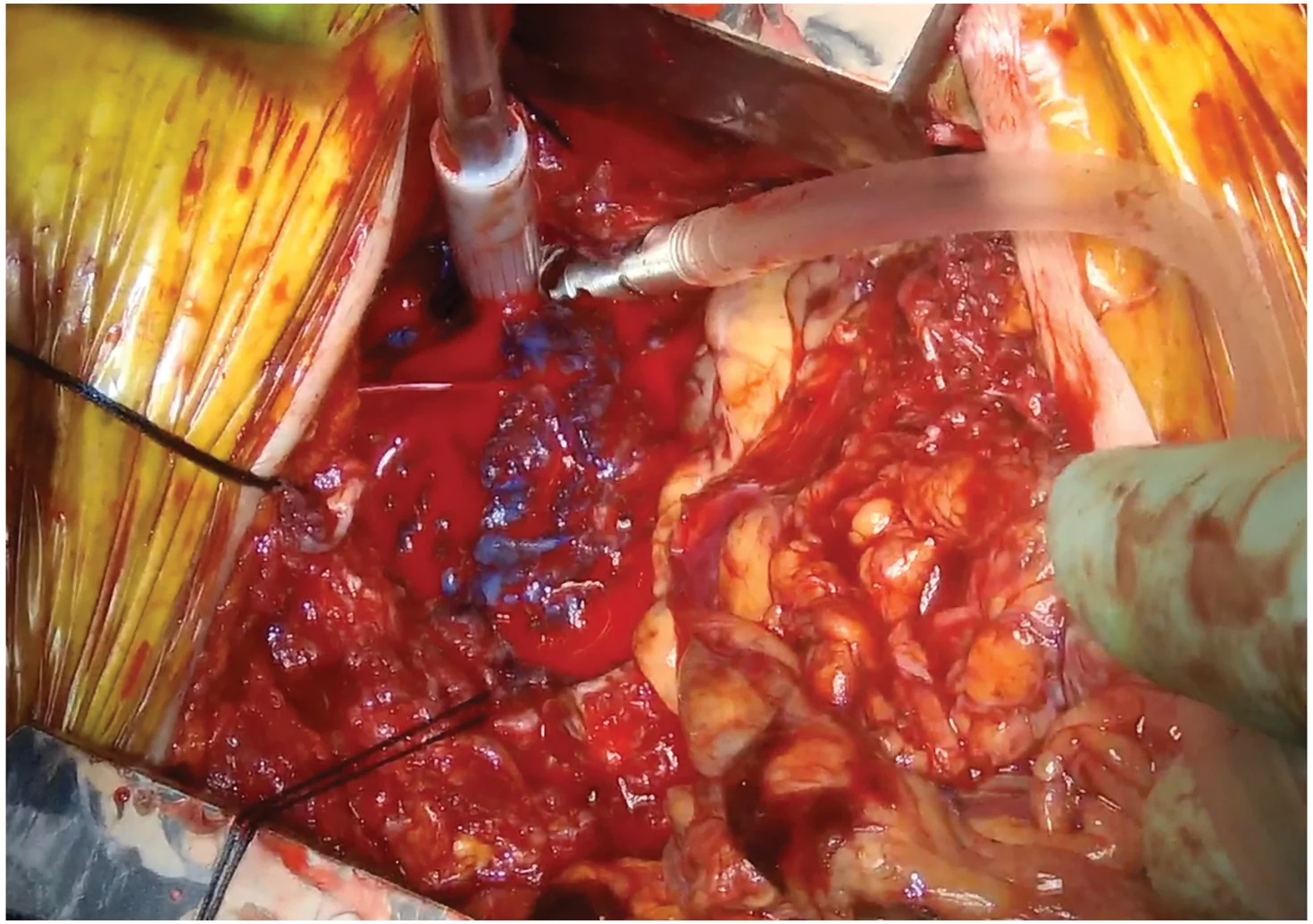
Active bleeding was observed from the aneurysmal area.

**Fig. 5:**
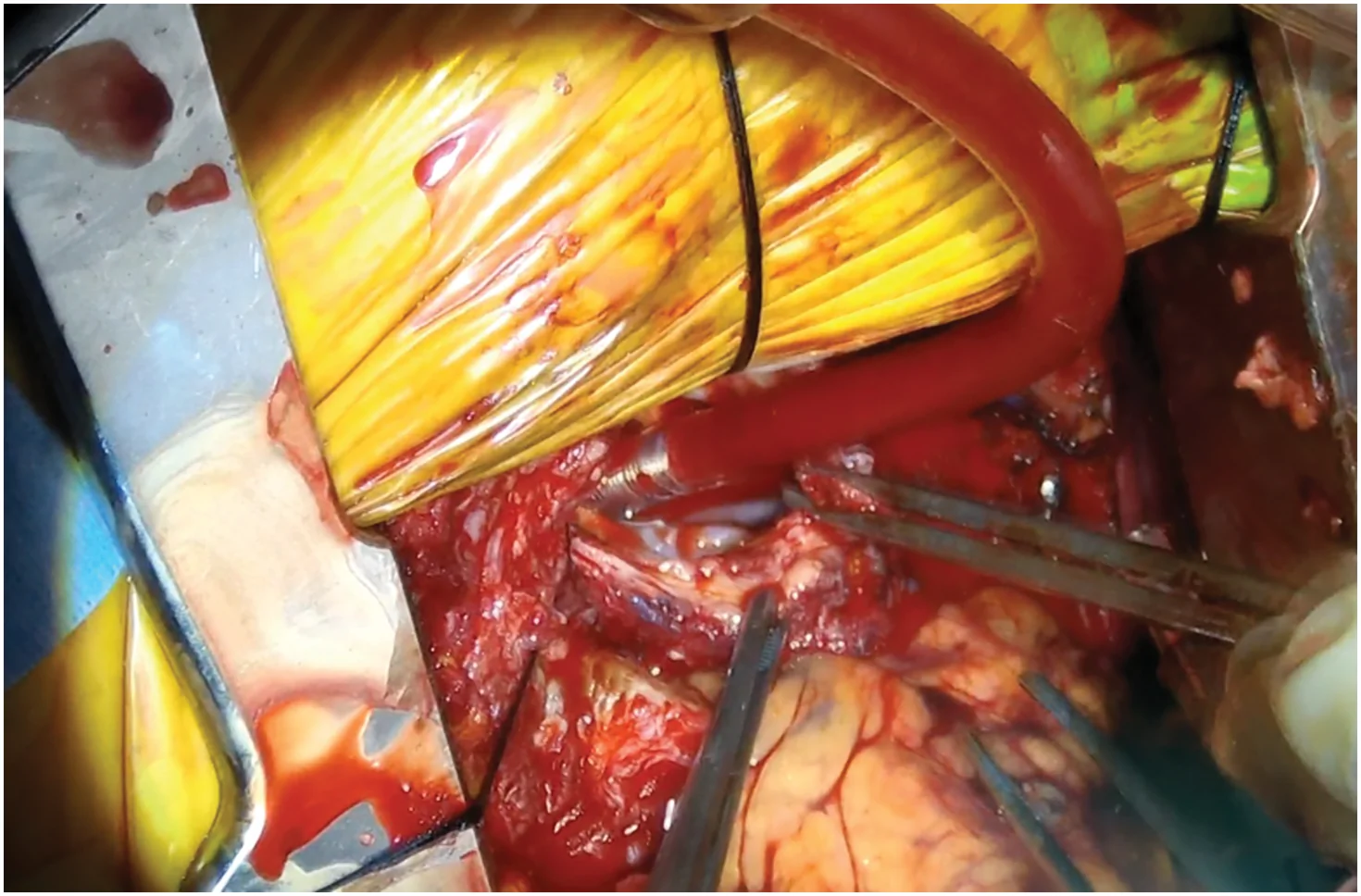
A 3 × 3-cm myocardial tissue defect was observed.

**Fig. 6:**
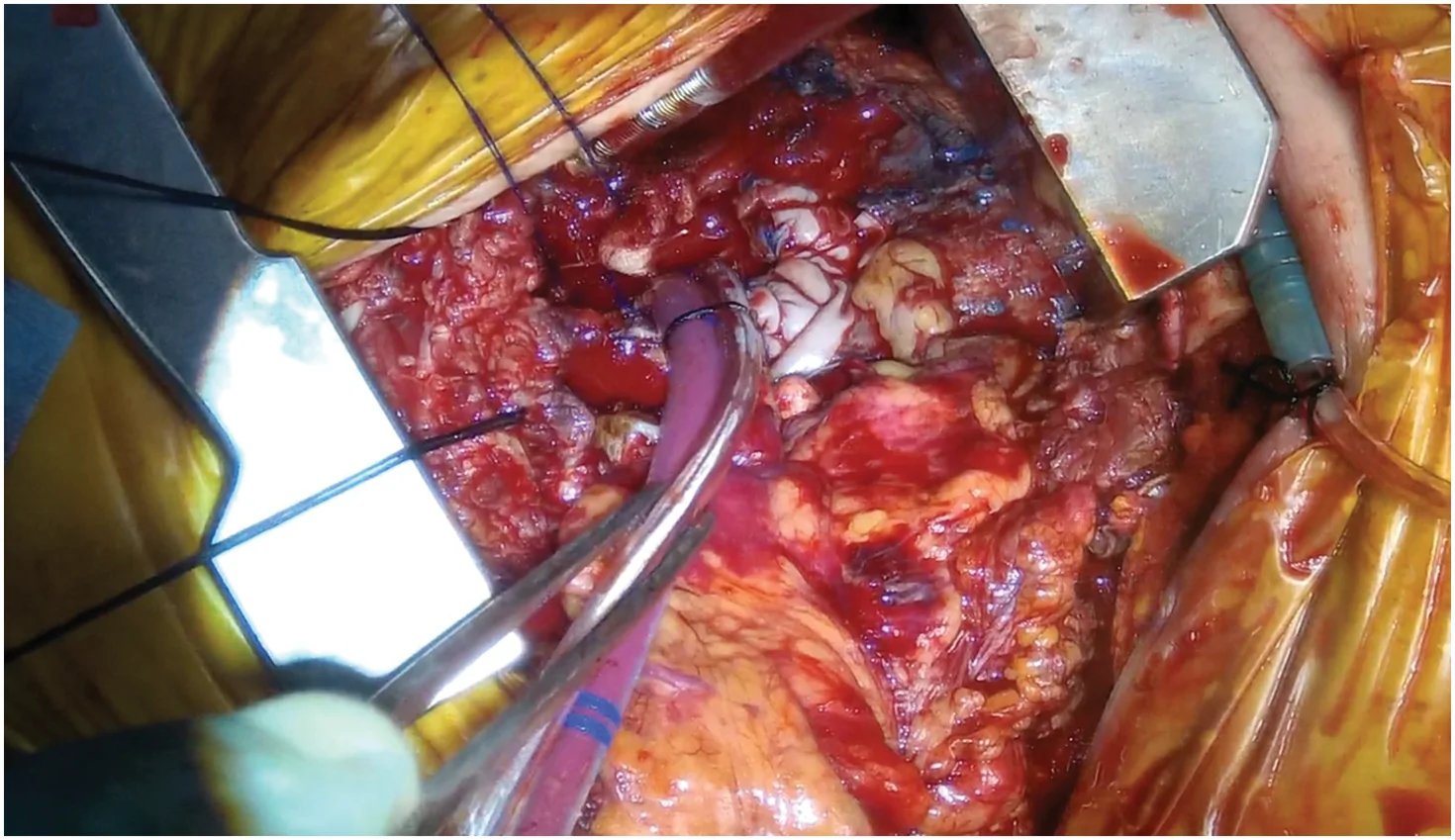
The defect was closed using a 4 × 4-cm bovine pericardial patch (Model 4700; Edwards Lifesciences, Irvine, CA, USA) with ten 2-0 MH U sutures (ETHIBOND EXCEL polyester suture, 2-0, MH needle, X843H; Ethicon, Somerville, NJ, USA). A left ventricular vent was inserted through the patch, and rewarming was then performed.

All intraoperative cultures, including those obtained from the excised bovine pericardial patch, were negative for infection. A histopathological examination of the fragile tissue excised from the aneurysmal site revealed an organizing hematoma without myocardial tissue. Postoperatively, she experienced prolonged respiratory failure, requiring tracheostomy and mechanical ventilation for 6 months. She was transferred to the rehabilitation ward but died 1 year after surgery due to pneumonia.

## DISCUSSION

An LVFWR occasionally occurs after acute myocardial infarction. The incidence of LVFWR has been reported to be 0.01%–0.5% in patients with acute myocardial infarction.^3)^ Medical treatment for LVFWR is normally unsuccessful, but surgical treatment for LVFWR has also been reported to have unsatisfactory outcomes. The postoperative in-hospital mortality has been reported to range from 32% to 36%.^1,2)^ According to the 2023 Japanese Association for Thoracic Surgery (JATS) annual report, surgery for a cardiac rupture was performed in 24 cases of chronic types and 271 cases of acute types. In-hospital mortality was reported to be 25% in chronic cases and 40.6% in acute ones.^4)^

The surgical strategy for cardiac rupture remains controversial. A re-rupture is a fatal postoperative complication that has been reported to occur in several cases,^1,2)^ typically during the initial hospitalization period. In a multicenter study involving 15 institutions,^1)^ a re-rupture occurred in 10 of 140 surgeries (7.1%), and the incidence was higher after a sutureless repair (11.1%) than after sutured repair (4.7%).^1)^ Other systematic reviews have also shown similar results, with a re-rupture occurring in 5.4% of sutureless repairs and in 1.7% of sutured repairs.

Reports on the long-term outcomes after surgery for LVFWR are limited. However, the 5-year survival rates range from 40% to 60%.^5,6)^ Specifically, for sutureless repair, Okamura et al. reported a 5-year survival rate of 68%.^7)^

As a late postoperative complication of a sutureless repair, a ventricular pseudoaneurysm has been reported in 12 cases in our PubMed search. The mean age of the patients was 62.9 years (range, 40–78 years), and the pseudoaneurysm developed at an average of 204 days (range, 7–810 days) after the initial surgery^8–19)^ (**Table 1**).

**Table 1:** Ventricular pseudoaneurysm after a sutureless repair

Age	Gender	Region	Type	Material for sutureless repair	Period since the first operation (day)	References
55	M	LCX	Blow out	TachoSil	7	^8)^
65	M	Diagonal Branch	Oozing	TachoSil	8	^9)^
71	F	RCA	Oozing	TachoSil, fibrin glue	8	^10)^
40	M	RCA	–	Bovine pericardium, Enbucrilate (Butyl cyanoacrylate)	30	^11)^
73	M	LCX	Oozing	TachoSil, fibrin glue, Bovine pericardium	49	^12)^
49	M	RCA	Oozing	BioGlue, PTFE Felt	60	^13)^
78	F	LCX	Oozing	TachoSil, autologous pericardium	90	^14)^
66	M	LAD	Oozing	TachoSil, fibrin glue, Bovine pericardium	120	^15)^
72	M	LAD	Oozing	TachoSil, autologous pericardium	150	^16)^
70	M	LCX	Oozing	GRF glue, Bovine pericardium	390	^17)^
51	F	LAD	Oozing	fibrin glue	730	^18)^
65	F	Diagonal Branch	Oozing+tear	GRF glue, autologous pericardium	810	^19)^

Summary of previously reported cases of ventricular pseudoaneurysm after a sutureless repair identified in a PubMed search. The mean of the patients was 62.9 years (range, 40–78 years), and the pseudoaneurysm developed at an average of 204 days (range, 7–810 days) after the initial surgery.

F, female; GRF, gelatin–resorcin–formalin; LAD, left anterior descending; LCX, left circumflex; M, male; PTFE, polytetrafluoroethylene; RCA, right coronary artery

This complication should therefore be considered an important issue during the postoperative follow-up. The present case was a blow-out-type cardiac rupture complicated by an anterior wall myocardial infarction, and it was repaired with the bovine pericardium as a sutureless repair. This case highlights that the tissue beneath a bovine pericardial patch cannot be assumed to have been replaced by stable fibrous tissue, especially when a ruptured myocardium is covered with bovine pericardial patches.

In the oozing-type rupture, when hemostasis can be achieved by compression with pericardial patches, then the infarcted myocardium will be replaced by fibrous tissue, which is expected to become the left ventricular aneurysmal wall. However, in the blow-out-type rupture, the formation of a hematoma just below the pericardial patches might occur. In the present case, an organizing thrombus was present and the myocardial tissue had disappeared just below the pericardial patches. It is hypothesized that a cleft develops within the infarcted myocardium, leading to blow-out cardiac rupture. Even when hemostasis is achieved by compression with pericardial patches and hematoma formation, hematoma may also develop within the myocardial cleft. As the hematoma progressively enlarges, the myocardial cleft is presumed to expand, and the resulting myocardial tissue defect is gradually replaced by an organizing hematoma (**Fig. 7**). This finding has important surgical implications. When removing the covered pericardial patches due to concerns about infection, a re-rupture must be anticipated. Careful evaluation of the underlying tissue is therefore mandatory, and surgeons should be prepared to convert to definitive repair if adequate tissue integrity is not present. Although the left thoracotomy provided adequate exposure, macroscopic findings alone could not reliably predict the fragility of the underlying tissue. Given the risk of unexpected re-rupture during patch removal, preparation for rapid initiation of CPB should be considered in similar cases.

**Fig. 7:**
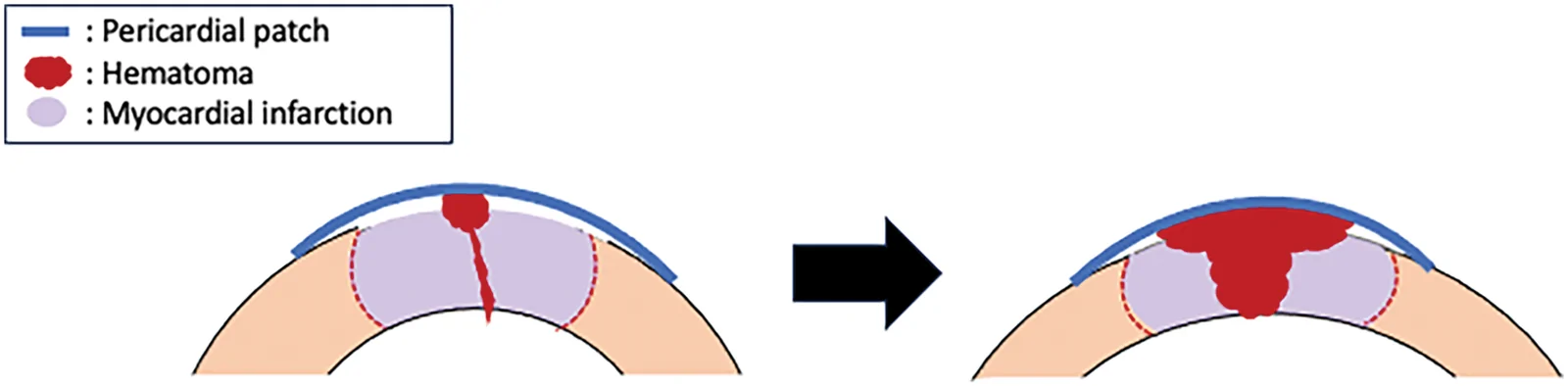
Proposed mechanism of myocardial rupture and subsequent remodeling in the blow-out-type. In the blow-out-type, a myocardial cleft develops within the infarcted myocardium, allowing blood to enter the subepicardial space from the ventricular cavity. Subsequent disruption of the epicardium results in bleeding. Even when hemostasis is achieved by compression with a pericardial patch and a hematoma is formed, hematoma formation may also occur within the myocardial cleft. As the hematoma progressively enlarges, the myocardial cleft is presumed to expand, and the resulting myocardial tissue defect is gradually replaced by an organizing hematoma. Left: Initial myocardial rupture and subepicardial bleeding. Right: Progressive hematoma formation and replacement of the myocardial defect.

In the present case, all intraoperative cultures were found to be negative. There were no obvious findings that passively suggested an infection in the initial pleural effusion. These results suggest that an impending rupture due to a pseudoaneurysm cannot be ruled out.

## CONCLUSIONS

Left ventricular pseudoaneurysm formation is a notable late complication that should be carefully monitored during the postoperative follow-up after surgery for LVFWR. We encountered a case of a re-ruptured left ventricular pseudoaneurysm after a sutureless bovine pericardial patch repair. The possibility of a re-rupture must be considered because the myocardial tissue beneath the pericardial patch might disappear and be replaced with an organizing hematoma.

## DECLARATIONS

### Availability of data and materials

Data sharing is not applicable to this article as no datasets were generated or analyzed during the current study.
